# Japanese Diet and Mortality, Disability, and Dementia: Evidence from the Ohsaki Cohort Study

**DOI:** 10.3390/nu14102034

**Published:** 2022-05-12

**Authors:** Sanae Matsuyama, Taichi Shimazu, Yasutake Tomata, Shu Zhang, Saho Abe, Yukai Lu, Ichiro Tsuji

**Affiliations:** 1 Division of Epidemiology, Department of Health Informatics and Public Health, Tohoku University School of Public Health, Graduate School of Medicine, Sendai 980-8575, Japan; s-matsuyama@med.tohoku.ac.jp (S.M.); lyk814@med.tohoku.ac.jp (Y.L.); 2 Division of Behavioral Sciences, Institute for Cancer Control, National Cancer Center, Tokyo 104-0045, Japan; tshimazu@ncc.go.jp; 3 School of Nutrition and Dietetics, Faculty of Health and Social Services, Kanagawa University of Human Services, Yokosuka 238-8522, Japan; toomata-5h0@kuhs.ac.jp; 4 Department of Epidemiology of Aging, Center for Gerontology and Social Science, National Center for Geriatrics and Gerontology, Obu 474-8511, Japan; zhangshu@ncgg.go.jp; 5 General Affairs and Human Resources Division, ROHTO Pharmaceutical Co., Ltd., Osaka 544-8666, Japan; sahoabe1011@gmail.com

**Keywords:** Japanese diet, mortality, disability, dementia, cohort study

## Abstract

The Japanese dietary pattern has long been discussed as one of the factors behind the longevity of Japanese people. However, the health benefits of the Japanese dietary pattern have not been fully elucidated. We published the first report in the world regarding the relation between the Japanese dietary pattern and cardiovascular disease mortality in 2007 using cohort studies including Japanese residents of Ohsaki City, Miyagi Prefecture, Japan. Since then, we have developed the Japanese Diet Index (JDI) that was based on previous findings to assess the degree of the Japanese dietary pattern and to advance the evidence on the health effects of the Japanese dietary pattern. So far, we have explored the associations between the JDI score (in quartiles) and various outcomes. For all-cause mortality, in comparison to Q1 (the lowest), the multivariable hazard ratios (HRs) and 95% confidence intervals (95%CIs) were 0.92 (0.85–1.00) for Q2, 0.91 (0.83–0.99) for Q3, and 0.91 (0.83–0.99) for Q4 (the highest). For functional disability, the multivariable HRs (95%CIs) were 0.94 (0.81–1.09) for Q2, 0.90 (0.77–1.05) for Q3, and 0.79 (0.68–0.92) for Q4. For dementia, the multivariable HRs (95%CIs) were 0.88 (0.74–1.05) for Q2, 0.87 (0.73–1.04) for Q3, 0.79 (0.66–0.95) for Q4. In addition, people with higher adherence to the Japanese dietary pattern also showed decreases in disability and dementia risks. The purpose of this article was to review all six papers, summarize the health effects of the Japanese dietary pattern, and discuss implications for future research.

## 1. Introduction

Why does the Japanese population not only have the longest life expectancy (84.3 years for both sex) but also have the longest healthy life expectancy (74.1 years for both sex) worldwide [1]? The Japanese dietary pattern has long been discussed as one of the factors behind the longevity of Japanese people [2]. However, the health benefits of the Japanese dietary pattern have not been fully elucidated, and with respect to this, in the Dietary Guidelines for Americans 2010 it is stated that “evidence is very limited” [3].

We published the first report in the world regarding the relation between the Japanese dietary pattern and cardiovascular disease (CVD) mortality in 2007. In this study, we prospectively followed a cohort of 40,547 Japanese people for seven years, and results showed that the Japanese dietary pattern had a significant association with decreased CVD mortality. [4] However, the Japanese dietary pattern used as the exposure in this study was simply one dietary pattern derived by factor analysis, so it was unclear whether greater conformity with the Japanese dietary pattern had an association with reduced CVD mortality. According to a previous review, the Japanese dietary pattern is usually demonstrated as a higher intake of soybean products, fish, vegetables, rice, seaweed, miso soup, pickles, and green tea [5]. Such foods as fish, and vegetables are in common with components included in the well-known healthy dietary patterns such as the Mediterranean diet [6], Dietary Approaches to Stop Hypertension (DASH) [7], and Healthy Eating Index (HEI) [8], which have been proven to be protective against a variety of chronic diseases [9,10,11]. Meanwhile, the Japanese dietary pattern also includes such unique components as seaweed, soybean, and green tea, each of which has been suggested to have potential benefits on health [12,13,14]. Thus, it is expected that the Japanese dietary pattern per se could play a critical role in health.

Our team has developed the Japanese Diet Index (JDI) including rice, miso soup, pickles, seaweeds, fish, green and yellow vegetables, green tea, beef and pork, and coffee, which is a priori diet index from predefined algorithms based on previous findings to quantify the adherence to the Japanese dietary pattern and to advance the evidence on its health benefits [15]. So far, we have published six articles regarding the relation between the JDI and such health outcomes as mortality, disability, and dementia [15,16,17,18,19,20]. In this article, we review all six papers, summarize the health benefits of the Japanese diet, and discuss possible implications for the future.

## 2. Materials and Methods

### 2.1. Development of the Japanese Diet Index

We have described the details of the JDI in a previous study [15]. In brief, nine food items were identified to form the JDI: rice, miso soup, pickles, seaweeds, fish, green and yellow vegetables, green tea, beef and pork, and coffee. These items were suggested to have higher absolute factor scores in the traditional Japanese dietary pattern [21], and were the main characteristic of the traditional Japanese diet [22].

For each of the seven adhering components (rice, miso soup, pickles, seaweeds, fish, green and yellow vegetables, and green tea), participants were assigned one point if their intake was above or equal to the sex-specific median. For each of the two non-adhering components (beef and pork, and coffee), participants were assigned one point if their intake was lower than the sex-specific median. The JDI score was generated by adding the scores in the nine components (0–9 points), and a higher score indicated higher adherence to the Japanese dietary pattern. Participants were categorized in quartiles, but the way of categorization in our studies was not the same due to differences in the number of study participants included in each study. For the associations with all-cause mortality and disability-free life expectancy, participants were categorized as Q1 (0–4), Q2 (5), Q3 (6), and Q4 (7–9) [16,18], while for the associations with functional disability and dementia, participants were categorized as Q1 (0–3), Q2 (4), Q3 (5), and Q4 (6–9) [15,19].

We then modified the JDI and developed the 8-item Japanese Diet Index (JDI8), by excluding coffee, which was included in the original JDI [23], as a previous meta-analysis has indicated that coffee intake may have benefits on reducing all-cause and CVD mortality [24].

### 2.2. Study Setting

#### 2.2.1. The Ohsaki Cohort 1994 Study (the 1994 Survey)

The Ohsaki Cohort 1994 Study was part of the Ohsaki National Health Insurance (NHI) Cohort Study, the detail of which was described in a previous study [25]. The NHI system covers all self-employed individuals, pensioners, and farmers and their dependents. The source population for the baseline survey comprised 54,996 individuals (40–79 years old), and all of them were NHI beneficiaries who lived in 14 municipalities within the catchment area of the Ohsaki Public Health Center, Miyagi Prefecture, Japan in 1994. In 2006, seven of fourteen municipalities were merged into the new Ohsaki City. Since then, the subjects of the seven municipalities (32,978 men and women in 1994) of the original Ohsaki NHI Cohort were followed in the name of the Ohsaki Cohort 1994 Study.

The 1994 survey was conducted from October to December 1994. Participants were asked to answer the questionnaires themselves and to return them within a week.

**Figure 1:**
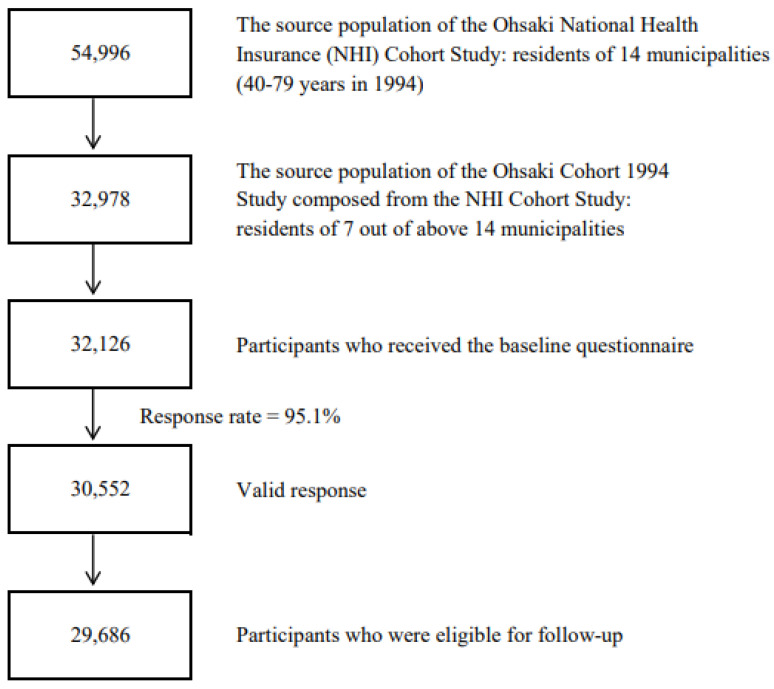
Flowchart of study participants of the Ohsaki Cohort 1994 Study.

In 1994, the questionnaires were distributed to 32,126 of 32,978 eligible individuals, and of those, 30,552 with valid responses formed the study cohort. Then, we excluded 60 individuals who had died or moved out before follow-up and 806 individuals not identified by the Residential Registry of Ohsaki City. A total of 29,686 participants were eligible for follow-up, which was from 1 January 1995, to 30 November 2019 (Figure 1).

#### 2.2.2. The Ohsaki Cohort 2006 Study (the 2006 Survey)

We have described the design in detail elsewhere [26]. The source population for the 2006 survey comprised all 31,694 older residents aged 65 years or older on 1 December 2006 living in Ohsaki City.

**Figure 2:**
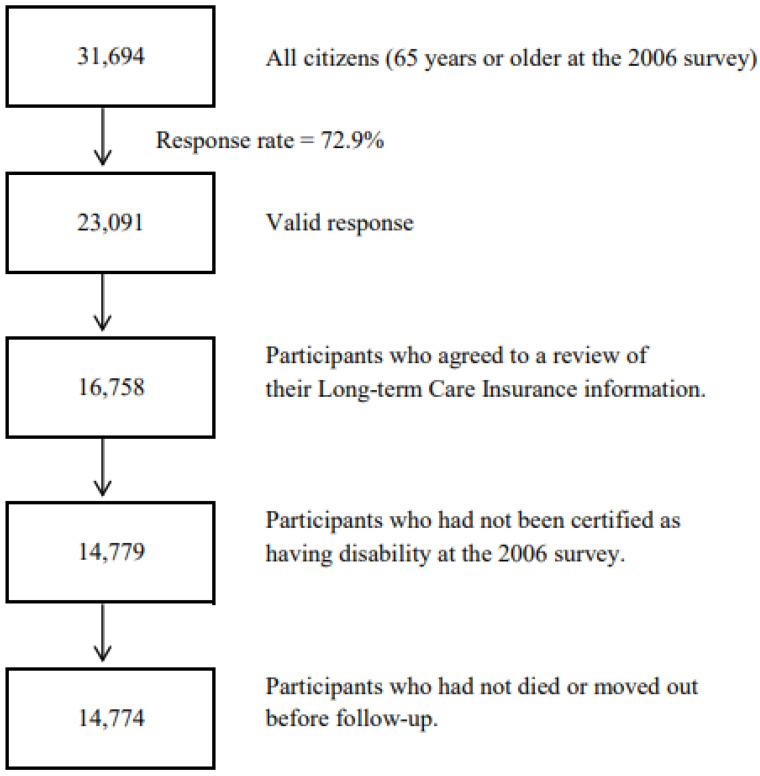
Flowchart of study participants of the Ohsaki Cohort 2006 Study.

The 2006 survey was conducted from 1 December 2006 to 15 December 2006. Of all the older citizens, 23,091 of them provided valid answers and formed the study cohort. We then excluded 6333 persons without written consent for review of their Long-term Care Insurance (LTCI) information, 1979 individuals who had been certified as disabled by the LTCI before the start of follow-up, and five individuals who had died or moved before the start of follow-up. A total of 14,774 individuals were eligible for follow-up, and the follow-up started from 16 December 2006, until 30 November 2019. (Figure 2).

### 2.3. Study Outcomes and Measurement

We examined three outcomes: all-cause death, disability, and dementia. The all-cause deaths were identified via the review of death certificates, with approval from the Ministry of Health, Labour, and Welfare of Japan and the Ministry of Internal Affairs and Communications of Japan.

Incident functional disability was defined as Support Level 1 or higher by LTCI certification. The LTCI is a mandatory form of national social insurance to assist activities of daily living among the disabled and older adults [27,28,29]. People aged ≥40 years paying premiums, and those aged ≥65 years are eligible for formal caregiving services under a uniform standard of disability certification [30]. The procedure for disability certification comprises two parts: assessment of the degree of functional disability using a questionnaire, and reference to the Doctor’s Opinion Paper prepared by the attending physician [31].

Incident dementia was defined as incident functional disability with dementia by the LTCI, using the Dementia Scale (Degree of Independence in Daily Living for Elderly with Dementia) entered on the Doctor’s Opinion Paper [32]. The Dementia Scale includes six ranks: 0, I–IV, and M. Rank M means severe behavioral and psychological symptoms requiring medical care. A rank ≥II is usually applied as the cutoff for definition of dementia [32,33,34]. It has been reported that the Dementia Scale showed a sensitivity of 73% and a specificity of 96% against clinical diagnoses [35].

A dataset was obtained annually in December, including information on the care level of LTCI certification, date of LTCI certification, date of death, and date of emigration from the Ohsaki City Government based on an agreement about the secondary use of data. The dataset was transferred under the agreement related to Epidemiologic Research and Privacy Protection.

### 2.4. Covariates and Measurement

Data on sociodemographic, lifestyle, and health-related factors were obtained in both 1994 and 2006. In detail, education level was measured with the question, “How old were you when you left school?” and we categorized participants as junior high school or less (<16 years), high school (16–18 years) or college or higher (≥19 years). Body mass index (BMI) was calculated as the self-reported body weight (kg) divided by the square of the self-reported body height (m), and participants were categorized into <18.5 kg/m^2^, 18.5–24.9 kg/m^2^, and ≥25.0 kg/m^2^. Time spent walking was assessed with the question, ‘How long on average do you walk per day?’, and participants were categorized into <0.5 h, 0.5–1 h or ≥1 h. Smoking status was divided as follows: never smokers; former smokers; and current smokers. Similarly, alcohol drinking status was divided as: never drinkers; former drinkers; and current drinkers. We asked participants whether they had suffered from the following diseases: hypertension; stroke, myocardial infarction; diabetes; osteoporosis; arthritis; falling/fracture; or cancer. In both 1994 and 2006, the consumption volume of each food item was calculated and energy intake and protein intake were estimated using a food composition table that corresponded to all items in the FFQ.

Psychological distress was only evaluated in 2006 by the Kessler 6-item Psychological Distress Scale [36,37]. Participants were asked about their mental status over the past four weeks with six questions (0–24 points). A score ≥ 13 was classified as psychological distress [37].

The Kihon Checklist was used in 2006, which was developed to predict functional decline in community-dwelling older adults. Regarding the motor function score in the Kihon Checklist, respondents were asked about their current motor function status with five questions (0–5 points). A score of <3 were classified as better motor function. Regarding the cognitive function score, respondents were asked about their current subjective memory complaints with three questions (0–3 points). A score of zero was classified as better cognitive function [38].

## 3. Results

### 3.1. Japanese Diet, Mortality, and Length of Survival

To investigate the associations between the JDI score and mortality and the length of survival, we used the setting of the Ohsaki Cohort 1994 Study (Study No. 1 in Table 1) [16]. A total of 14,764 participants were categorized in quartiles (Q1: 0–4 points [*n* = 4782]; Q2: 5 points [*n* = 3243]; Q3: 6 points [*n* = 3219]; Q4: 7–9 points [*n* = 3520]).

Multivariable Cox proportional hazard models were applied to estimate the hazard ratios (HRs) and 95% confidence intervals (CIs) for all-cause mortality, which adjusted for sex, education level, alcohol drinking, smoking, time spent walking, body mass index, history of diseases, and energy intake, using age as the time scale. Results showed that higher JDI scores were related to lower all-cause mortality. In comparison to participants in Q4 (the lowest), the multivariable HRs (95% CIs) for all-cause mortality were 0.92 (0.85–1.00) in Q2, 0.91 (0.83–0.99) in Q3, and 0.91 (0.83–0.99) in Q4 (Table 2).

We performed Laplace regression for estimating differences in survival time between the quartiles of the JDI score [16]. Laplace regression model estimates the percentile of the time variable of concern [39,40]. Differences in median (50th PDs) age at death and their 95% CIs were calculated. Results suggested that people with higher JDI scores had a longer survival time. The multivariable 50th PDs (95% CIs) of age at death were 8.9 (2.6–15.2) months longer in Q2, 10.4 (3.4–17.3) months longer in Q3, and 10.2 (3.2–17.2) months longer in Q4, in comparison with participants in Q1 (the lowest) (Table 2).

### 3.2. Japanese Diet, Disability, and the Length of Disability-Free Survival

First, we examined the association between the JDI score and incident functional disability, using the setting of the Ohsaki Cohort 2006 Study (Study No. 2 in Table 1) [15]. 10,148 individuals were categorized in quartiles (Q1: 0–3 points [*n* = 2247]; Q2: 4 points [*n* = 2096]; Q3: 5 points [*n* = 2400]; Q4: 6–9 points [*n* = 3405]). Multivariable Cox proportional hazard models were applied to estimate the HRs (95%CIs) for incident functional disability, which were adjusted for sex, age, education level, alcohol drinking, smoking, time spent walking, body mass index, history of diseases, psychological distress, motor function, energy intake and protein intake. Results suggested that people in a higher quartile of the JDI score showed lower incident risk of functional disability. The multivariable HRs (95% CIs) for incident functional disability were 0.94 (0.81–1.09) in Q2, 0.90 (0.77–1.05) in Q3, and 0.79 (0.68–0.92) in Q4, in comparison with Q1 (Table 2).

Second, the settings of both the Ohsaki Cohort 1994 Study and the Ohsaki Cohort 2006 Study were applied to investigate the effect of changes in the JDI score on incident risk of functional disability (Study No.3 in Table 1) [17]. We categorized a total of 2923 individuals into five groups according to changes in the JDI score between 1994 and 2006 (great decrease [≤–2 points, *n* = 462], moderate decrease [–1 point, *n* = 513], no change [=0, *n* = 648], moderate increase [+1 point, *n* = 642], and great increase [≥+2 points, *n* = 658]). Multivariable Cox proportional hazard models were applied to estimate the HRs (95%CIs) for incident functional disability, which were adjusted for sex, age, education level, alcohol drinking, smoking, time spent walking, body mass index, history of diseases, psychological distress, and energy intake in 2006 and smoking, alcohol drinking, time spent walking, body mass index, history of disease, the JDI score and energy intake in 1994. Results showed that participants with an increase in the JDI score had a decreased risk of functional disability. Compared to a great decrease (≤–2 points), the multivariable HRs (95% CIs) for incident functional disability were 0.82 (0.66–1.01) for moderate decrease, 0.80 (0.65–0.98) for no change, 0.76 (0.61–0.95) for moderate increase, and 0.77 (0.61–0.98) for great increase (Table 2).

Third, we also examined the relation of the JDI score to disability-free survival time, using the Ohsaki Cohort 2006 Study (Study No. 4 in Table 1) [18]. A total of 9456 participants were categorized in quartiles (Q1: 0–4 points [*n* = 3426]; Q2: 5 points [*n* = 2009]; Q3: six points [*n* = 1983]; Q4: 7–9 points [n = 2038]). Laplace regression was performed to calculate differences in median age at disability or death (50th PDs in age at incident disability or death) and their 95% CIs, adjusted for sex, age, education level, alcohol drinking, smoking, time spent walking, body mass index, history of diseases, psychological distress, number of remaining teeth, motor function, cognitive function, energy intake and protein intake. Results indicated that people with greater JDI scores showed longer disability-free survival time. In comparison to people in Q1, the disability-free survival time were 2.0 (–2.7–6.8) months longer in Q2, 5.9 (1.4–10.3) months longer in Q3, and 7.1 (1.8–12.4) months longer in Q4 (Table 2).

### 3.3. Japanese Diet and Dementia

We examined the relation between the JDI score and dementia, using the setting of the Ohsaki Cohort 2006 Study (Study No.5 in Table 1) [19]. We categorized 14,402 participants in quartiles (Q1: 0–3 points [*n* = 2921]; Q2: 4 points [*n* = 2644]; Q3: 5 points [*n* = 3130]; Q4: 6–9 points [*n* = 5707]). Multivariable Cox proportional hazard models were applied to estimate the HRs 95%CIs for incident dementia, which were adjusted for sex, age, education level, alcohol drinking, smoking, time spent walking, body mass index, history of diseases, psychological distress, number of remaining teeth, motor function, cognitive function, energy intake and protein intake. Results suggested that the JDI score had an inverse association with incident risk of dementia. Compared to Q1, the multivariable HRs (95% CIs) for incident dementia were 0.88 (0.74–1.05) in Q2, 0.87 (0.73–1.04) in Q3, and 0.79 (0.66–0.95) in Q4 (Table 2).

In addition, to investigate the effect of changes in the JDI8 score on incident dementia, we used both the Ohsaki Cohort 1994 Study and the Ohsaki Cohort 2006 Study (Study No. 6 in Table 1) [20]. A total of 3146 individuals were divided into five groups according to changes in the JDI8 score (great decrease [≤–2 points, *n* = 584], moderate decrease [–1 point, *n* = 599], no change [=0e, *n* = 784], moderate increase [+1 point, *n* = 635], and great increase [≥+2 points, *n* = 544]). Multivariable Cox proportional hazard models were applied to estimate the HRs 95%CIs for incident dementia, which were adjusted for sex, age, education level, alcohol drinking, smoking, time spent walking, body mass index, history of diseases, psychological distress, and energy intake in 2006 and alcohol drinking, smoking, time spent walking, body mass index, history of diseases, the JDI8 score, and energy intake in 1994. Results showed that people with an increase in the JDI8 score from 1994 to 2006 showed decreased risk of incident dementia. In comparison with no change group, the multivariable HRs (95% CIs) for incident dementia was 1.72 (1.13–2.62) in great decrease, 1.10 (0.73–1.66) in moderate decrease, 0.82 (0.54–1.25) in moderate increase, and 0.62 (0.38–1.02) in great increase (Table 2).

## 4. Discussion

Our findings have demonstrated that the Japanese dietary pattern has association with multiple health outcomes, such as longer survival and lower risks of disability and dementia. The Japanese dietary pattern was also significantly related to reduced CVD mortality. In Japan, both incident disability and dementia are largely due to CVD, so we consider that the reduction in CVD risk and the reductions in incident disability and dementia risk are consistent.

As these studies were observational, we cannot determine the causality of the Japanese dietary pattern with mortality, disability, and dementia. However, we also reported that people with an increase in the JDI score showed decreased risks of disability and dementia; thus, this may suggest a causal relationship of the Japanese dietary pattern with health benefits.

Various beneficial components are contained in the Japanese dietary pattern such as vegetables, fish, miso (made from soybean), seaweeds, pickles, and green tea. These unique foods may have helped reduce the risks of mortality, disability, and dementia, as it has been reported that these foods are related to decreased risks of all-cause and CVD mortality, CVD morbidity, incident disability, cognitive decline, and dementia [41,42,43,44,45,46,47]. In addition, the healthy foods that compose the Japanese dietary pattern include various beneficial nutrients, for instance, vegetables contain potassium, carotenoids, vitamin C, fiber, antioxidants, and flavonoids [48,49], fish contain DHA, EPA, and very-long-chain fatty acids [50,51,52], soybean products contain isoflavane [53,54], seaweeds contain dietary fiber [44], and green tea contains polyphenol catechins with antioxidative activity [30,55]. These nutrients have been reported to have preventive effects on non-communicable diseases that may cause disability and dementia. Therefore, it is considered that the reductions in risks of mortality, disability, and dementia were related to not only individual foods, but also the Japanese dietary pattern as a whole.

However, there is a concern about the higher amount of sodium contained in the Japanese dietary pattern. Regarding this issue, a previous study reported that people with greater adherence to the Japanese dietary pattern consumed not only sodium, but also such beneficial nutrients as protein, calcium, iron, magnesium, potassium, vitamins A, C, and E, and fiber [23,56]. Therefore, in the Japanese dietary pattern, the adverse health effects of sodium may be counteracted by other beneficial nutrients.

## 5. Implications for Future Research

So far, our studies have revealed associations between the Japanese dietary pattern and various health outcomes and consistently reported potential health benefits of the Japanese dietary pattern. These findings may be partly attributable to the cumulative effects of individual food items of the Japanese dietary pattern. Due to the complex biological interactions between various components included in the Japanese dietary pattern, using a whole diet approach instead of individual food groups or nutrients may contribute to understanding the role played by the Japanese dietary pattern in health and longevity [57].

Since our JDI was established in a region of northeast Japan, Ohsaki City, its generalizability must be verified. We have already examined the association between the JDI8 and mortality using the Japan Public Health Center-based Prospective Study dataset, participants of which were from 11 public health center areas in Japan [7]. A total of 92,969 participants were categorized in quartiles of the JDI8 score (Q1: 0–2 points [*n* = 16,838]; Q2: 3 points [*n* = 15,461]; Q3: 4–5 points [*n* = 36,196]; Q4: 6–8 points [*n* = 24,474]). Results suggested that people with a higher JDI8 score showed lower all-cause mortality. The multivariate-adjusted HRs (95% CIs) for all-cause mortality were 0.95 (0.90–0.99) in Q2, 0.91 (0.87–0.95) in Q3, and 0.86 (0.81–0.90) in Q4, compared to Q1. This result suggests that our findings may be generalizable to the whole Japanese population.

In addition, we believe that interventional studies remain a critical and irreplaceable tool in elucidating the health effects of the Japanese diet. Several interventional studies have been conducted on the Mediterranean diet, which is known to be a healthy diet. To promote discussions that can include the drawing of causal relationships between diet and health outcomes, it is necessary to conduct interventional studies in addition to observational studies.

## Figures and Tables

**Table 1:** Outline of published articles.

Study No.	First Author	Study Setting	Exposure	Outcome	Reference No.
1	Abe et al.	The Ohsaki Cohort 1994 Study	The JDI score	Mortality and survival time	16
2	Tomata et al.	The Ohsaki Cohort 2006 Study	The JDI score	Incident functional disability	15
3	Matsuyama et al.	The Ohsaki Cohort 1994 and 2006 Study	Changes in the JDI score	Incident functional disability	17
4	Zhang et al.	The Ohsaki Cohort 2006 Study	The JDI score	Disability-free survival time	18
5	Tomata et al.	The Ohsaki Cohort 2006 Study	The JDI score	Incident dementia	19
6	Lu et al.	The Ohsaki Cohort 1994 and 2006 Study	Changes in the JDI8 score	Incident dementia	20

JDI: Japanese Diet Index, JDI8: 8-item Japanese Diet Index.

**Table 2:** Summary of published results.

the JDI Score	Q1 (0–4)	Q2 (5)	Q3 (6)	Q4 (7–9)		*p*-Trend
**All-cause mortality/Survival time (*n* = 14,764)**						
No. of participants	4782	3243	3219	3520		
No. of events	1388	949	1049	1233		
Person years	82,425	55,808	55,316	59,708		
HRs (95%CIs) ^a,b^	1.00 (reference)	0.92 (0.85–1.00)	0.91 (0.83–0.99)	0.91 (0.83–0.99)		0.027
50th PDs (95%CIs) ^c,d^	0.00 (reference)	8.9 (2.6–15.2)	10.4 (3.4–17.3)	10.2 (3.2–17.2)		0.007
	**Q1 (0–3)**	**Q2 (4)**	**Q3 (5)**	**Q4 (6–9)**		
**Functional disability (*n* = 10,148)**						
No. of participants	2247	2096	2400	3405		
No. of events	374	333	374	481		
Person years	9793	9293	10,661	15,261		
HRs (95%CIs) ^a,e^	1.00 (reference)	0.94 (0.81–1.09)	0.90 (0.77–1.05)	0.79 (0.68–0.92)		0.002
	**Q1 (0–4)**	**Q2 (5)**	**Q3 (6)**	**Q4 (7–9)**		
**Disability-free survival time (*n* = 9456)**						
No. of participants	3426	2009	1983	2038		
No. of events	1564	902	892	875		
Person years	26,110	15,567	15,379	16,125		
50th PDs of DFS (95%CIs) ^f,g^	0.00 (reference)	2.0 (−2.7–6.8)	5.9 (1.4–10.3)	7.1 (1.8–12.4)		<0.001
	**Q1 (0–3)**	**Q2 (4)**	**Q3 (5)**	**Q4 (6–9)**		
**Dementia (*n* = 14,402)**						
No. of participants	2921	2644	3130	5707		
No. of events	314	249	263	463		
Person years	13,976	13,001	15,456	28,611		
HRs (95%CIs) ^a,h^	1.00 (reference)	0.88 (0.74–1.05)	0.87 (0.73–1.04)	0.79 (0.66–0.95)		0.015
**Changes in the JDI score**	**Great decrease (≤−2)**	**Moderate decrease (=−1)**	**No change (=0)**	**Moderate increase (=+1)**	**Great increase (≥+2)**	
**Functional disability (*n* = 2923)**						
No. of participants	462	513	648	642	658	
No. of events	191	191	238	228	245	
Person years	3321	3810	5102	5055	5178	
HRs (95%CIs) ^a,i^	1.00 (reference)	0.82 (0.66–1.01)	0.80 (0.65–0.98)	0.76 (0.61–0.95)	0.77 (0.61–0.98)	0.045
**Changes in the JDI8 score**	**Great decrease (≥−2)**	**Moderate decrease (=−1)**	**No change (=0)**	**Moderate increase (=+1)**	**Great increase (≥+2)**	
**Dementia (*n* = 3146)**						
No. of participants	584	599	784	635	544	
No. of events	58	43	56	42	32	
Person years	2767	2970	3995	3242	2783	
HRs (95%CIs) ^a,j^	1.72 (1.13–2.62)	1.10 (0.73–1.66)	1.00 (reference)	0.82 (0.54–1.25)	0.62 (0.38–1.02)	<0.0001

JDI: Japanese Diet Index, JDI8: 8-item Japanese Diet Index, HR: hazard ratio, 50th PD: 50th percentile difference, 95% CI: 95% confidence interval, DFS: disability-free survival. ^a^ HRs (95% CIs) were calculated by Cox proportional hazards models. ^b^ Adjusted for sex, education level, alcohol drinking, smoking, time spent walking, body mass index, history of diseases, and energy intake, with age as the time scale. ^c^ The 50th PD was defined as differences in age at median survival time in month compared to the reference group and calculated by Laplace regression. ^d^ Adjusted for sex, age, education level, alcohol drinking, smoking, time spent walking, body mass index, history of diseases, and energy intake. ^e^ Adjusted for sex, age, education level, alcohol drinking, smoking, time spent walking, body mass index, history of diseases, psychological distress, motor function, energy intake and protein intake. ^f^ The PD was defined as differences in age at median survival time without disability in a month compared to the reference group and calculated by Laplace regression. ^g^ Adjusted for sex, age, education level, alcohol drinking, smoking, time spent walking, body mass index, history of diseases, psychological distress, motor function, and energy intake. ^h^ Adjusted for sex, age, education level, alcohol drinking, smoking, time spent walking, body mass index, history of diseases, psychological distress, number of remaining teeth, motor function, cognitive function, energy intake and protein intake. ^i^ Adjusted for sex, age, education level, alcohol drinking, smoking, time spent walking, body mass index, history of diseases, psychological distress, and energy intake in 2006 and smoking, alcohol drinking, time spent walking, body mass index, history of diseases, the JDI score and energy intake in 1994. ^j^ Adjusted for sex, age, education level, alcohol drinking, smoking, time spent walking, body mass index, history of diseases, psychological distress, and energy intake in 2006 and alcohol drinking, smoking, time spent walking, body mass index, history of diseases, energy intake, and the JDI8 score in 1994.

## Data Availability

Data described in the manuscript, code book, and analytic code will not be made publicly available because private information of participants was included but are available from the corresponding author on reasonable request.

## Abbreviations

CI	confidence interval
CVD	cardiovascular disease
HR	hazard ratio
JDI	Japanese diet Index
JDI8	8-item Japanese Diet Index
LTCI	Long-term Care Insurance
NHI	National Health Insurance
